# Chemistry and Vegetable Food

**Published:** 1850-10

**Authors:** 


### ARTICLE XVII.

### *Chemistry and Vegetable Food.*

We abridge, from the *Edinburg Review*, the following important observations relative to this scientific subject, regretting that our space will not permit our giving the entire article:

Recent determinations of the proportions of nitrogen in wheat have served to correct erroneous opinions. It was long believed that the wheat of warm climates, as containing more nitrogen, was more nutritive and pecuniarily valuable than the wheat of temperate countries. But late researches show British wheat to be economical and nutritive as compared with the wheat of India, Australia or the Black Sea. The British miller seeks foreign wheat to mix with British grain, for ease in grinding, to produce for the baker a flour that will stand more water. The pastry cook and maccaroni maker need a flour to make peculiarly adhesive dough. These were supposed to be qualities inherent only to wheat abounding in gluten. Modern chemistry corrects the mistake. So in regard to muscle-forming matter which was supposed to exist more in wheat than in oats. But experience has shown that oats such as are grown in Scotland form most nutritious food. English habits, and the celebrated Dr. Johnson's ridicule, had nearly shamed the Scotch out of their national diet; but chemistry steps in to vindicate it, and shows that oats and oatmeal excel, as muscle-forming matter, the best wheat and the finest wheaten flour. Chemistry has further shown the value of the parts heretofore treated as waste. The husk or bran is proved by analysis to be richer in muscular matter than the white interior of the grain, which explains the cause of its answering so well as food for cattle. The true value of other food is also established by these inquiries. Cabbage has hitherto not been a general favorite in this country, either in the stall or for the table. In North Germany and Scandinavia it has long been esteemed. The result of recent examination, suggested by the failure of potatoes, is both interesting and unexpected. When dried so as to become fairly compared with wheat, oats, beans, &c., it is found richer in muscular matter than any other crop we grow. Wheat contains but 12 per cent., beans 25; but cabbage gives 40 per cent. of the so-called protein compounds. It is therefore pre-eminently nourishing. Hence, if it can be but made agreeable to the palate and easy of digestion, it is likely to prove the best substitute for the potato. Irish kolcannon, (cabbage and potatoes beaten together,) derives its fame from the more muscular sustaining power of the cabbage, in which the potato is deficient. An acre of ordinary land will produce a greater weight of this special kind of nourishment than in any other crop. But cabbage is so exhausting to many soils that wheat will scarcely produce an abundant crop after it. Such wheat springs up, but yields little straw, and quickly runs to a puny ear, containing little grain. But the chemical analysis points out the means of repairing this exhaustion at the least cost. In many parts of England furze or gorse grows an unheeded weed, and luxuriates in vain. In other districts large breadths of it are grown for feeding stock, and yield profitable returns. Chemical research proves its nutritive value. Of muscle-forming materials it contains, when dry, 30 per cent., and is inferior herein only to the cabbage. No doubt can, therefore, be entertained that the more extensive cultivation of gorse on poor soils would be most advantageous.

The Tussac grass, a native of the Falkland Islands, is most fattening for stock, especially for horses. Some seeds thereof have been lately imported, and the products of its vegetation in this country have been analysed. The proportion of muscle-forming ingredients in the dried grass is as great as in the best samples of wheat, oats or barley. Thus its feeding qualities are confirmed, and we may look to see it domesticated in Great Britain. The money value of the above investigations need not be dilated upon, but the results indicate questions of a higher kind, which will readily occur to the reader.

